# Exploring gender, age, time and space in research with older Pakistani Muslims in the United Kingdom: formalised research ‘ethics’ and performances of the public/private divide in ‘the field’

**DOI:** 10.1017/S0144686X14001378

**Published:** 2015-02-11

**Authors:** MARIA ZUBAIR, CHRISTINA VICTOR

**Affiliations:** * School of Sociology and Social Policy, University of Nottingham, UK.; † Centre for Dementia, Institute of Mental Health, University of Nottingham, UK.; ‡ College of Health and Life Sciences, Brunel University London, UK.

**Keywords:** ethnicity, fieldwork, older people, public/private, research ethics

## Abstract

In recent years, there has been an increasing interest in researching ageing ethnic minority populations in the West. However, older people from such minority communities have received comparatively little attention in wide-ranging discussions on appropriate research methodologies. By a process of critically reflecting on our experiences of undertaking fieldwork for our Economic and Social Research Council New Dynamics of Ageing study of ‘Families and Caring in South Asian Communities', this paper maps out the key methodological and ethical challenges we faced and, in the process, highlights the importance of developing socially appropriate research methodologies and ethical frameworks for research with such populations. With a reflexive approach, we specifically explore the significance of gender, age, time and space to the fieldwork processes and the ‘field’ relationships formed at various stages of the research process. In particular, we explore three key emergent issues which conflicted with our formal research protocols and presented particular challenges for us and our older Pakistani Muslim participants: (a) structuring of time in daily life; (b) gendered use of public and private spaces; and (c) orality of informal social contexts and relationships. Using illustrations from our fieldwork which reveal the particular significance of these issues to our fieldwork experiences and performativities of public/private identities, we highlight important tensions between formalised ethical and methodological dimensions of conducting funded research and the realities of being in ‘the field’. We conclude the paper by emphasising the need to explore further not only the ways in which researchers can adopt more socially and culturally sensitive data collection processes and methodologies at the micro level of their interactions with research participants, but also contextualising the particular challenges experienced by researchers and their participants in terms of the wider research frameworks and agendas as well as the broader social contexts within which they live and work.

## Introduction

The recent demographic changes relating to the increasing numbers of older people from ethnic minority backgrounds ageing in their Western host countries (Burholt 2004; Jimenez *et al.* 2012; Victor, Martin and Zubair 2012) have led to a greater need and interest in researching these older minority populations (Victor, Martin and Zubair 2012; Vincent, Phillipson and Downs 2006). However, in just the same way as with research with older people more generally (*see* Gledhill, Abbey and Schweitzer 2008; Greenwood 2009; Jaffe and Miller 1994; Kayser-Jones and Koenig 1994; Locher *et al.* 2006), research with ethnic minority older people has been perceived within most of the existing literature as being fraught with particular practical, methodological and ethical challenges (*see* Bowes and Dar 2000; Curry and Jackson 2003; Feldman *et al.* 2008; Fitzpatrick *et al.* 2012; Lichtenberg *et al.* 2004; Low *et al.* 2009).

Given the perceived ethical, methodological and procedural challenges of conducting research with older people, and especially ethnic minority older people, an increasing body of the methodological literature has attempted to identify appropriate research methods and procedures for both researching ethically and at the same time enhancing fieldwork with these populations (*see* Arean *et al.* 2003; Chadiha *et al.* 2004; Levkoff and Sanchez 2003; Shearer, Fleury and Belyea 2010; Sugarman, McCrory and Hubal 1998; Wenger 2002). This methodological literature has positioned these older populations predominantly in terms of their perceived social and cultural differences from younger populations more generally, presuming homogeneity within distinct age- and ethnicity-based social categories or groupings. Furthermore, this literature has shown a tendency towards locating the fieldwork challenges of researching these populations in these populations' own particular age- and ethnicity-based differences, thus implicitly constructing these populations in terms of their deviation from the standardised norms of participation expected of the more ‘easily researched’ social groups.

Noting the distinctive ethical and methodological requirements which have traditionally been proposed in relation to research with older people who are often defined stereotypically in terms of their vulnerability, Russell (1999: 414) observes that ‘concepts like vulnerability should not uncritically be transferred to an analysis of the research act’. Pointing to the different ways in which her own older research participants – even though socially isolated and in need of support services – exercised considerable power over the course of her research, she challenges the dominant conceptualisations of older people as ‘vulnerable subjects' who may be easily coerced and exploited within the research process. As Russell further observes, her emphasis on the older participants as being equally active agents within the research process is not to suggest that the problems of ageing or the vulnerabilities of older people are not real or significant and hence need not be taken into account in the ethical and methodological considerations relating to the research process. On the contrary, given that the specific agendas and foci of research with respect to older populations and ageing have traditionally been connected with a concern for the provision of these older populations' health and social care needs, research in the field of later life and ageing has mainly involved older people in vulnerable and socially marginalised positions. Such earlier research agendas and foci have, however, had consequences in terms of the almost universal social construction and perception of ‘older people’ as a homogenised vulnerable social group or category, as noted by Leontowitsch (2012: 1):

…largely due to a political economy perspective that focused on poverty and ageing as a residual category … research was geared to measuring need and assessing ways in which health and social care could meet these in an economic way. Although the political economy focus has provided valuable insights into the plight of older people (and predominantly older women), it has led to viewing older people as a homogeneous group who live in deprived circumstances. The economic focus has been met by a biomedical one, which depicts ageing as a biological and inevitable downward trajectory of physical decline. Thus older people have been regarded as passive recipients of this economic and biological plight.

While older people more generally continue to be perceived and treated in Western research, social policies, media and society at large as an undifferentiated social grouping which is vulnerable, frail, poor and in need of assistance (Leontowitsch 2012; Russell 1999), ethnic minority older people in particular have become subjected to Western research, social policy and wider public discourses which are concerned with their specific ‘difference’, and which construct them as the homogeneous *problematic Others* with their own further ‘special needs' as immigrants (Sin 2004; Torres 2006). This pathological construction of both old age and minority ethnic status, and the corresponding perception of older ethnic minority people as the ageing ethnic *Others* with special needs, also extends to researchers' use of formalised ethical guidelines and protocols in conducting research with these social groups. However, rather than introducing greater sensitivity within the research context, as Sin (2004: 268) rightly argues, this public discourse of ‘special needs', ‘deprivation’ (and ‘vulnerability’) commonly used within research with older ethnic minority people reinforces the power imbalances between the researcher and the researched. This is because such a discourse undermines the personal achievements and individuality of the ethnic minority older people (*see also* Blakemore and Boneham 1994), and can therefore be disempowering for these research participants as opposed to being more ethical.

More recently, there has been a small move towards the use of participatory approaches in ageing research more generally, whereby older people have been closely involved in research as active co-participants defining and guiding the research process (*see* Blair and Minkler 2009; Chambers and Pickard 2001; Cook, Maltby and Warren 2005; Warren *et al.* 2003).^1^ Such participatory approaches tend to negate the ‘othering’ of older participants and disrupt, to some extent, the insider/outsider and public/private divides and hierarchies that otherwise prevail within the research process. While such research approaches may be seen as empowering the older research participants and hence more ethical, as Walker (2007) notes, there are very few examples of their use to learn from.

With respect to research on ethnic minority populations, including research on ethnic minority older people, the predominant means of addressing the power imbalances and the ethical and methodological challenges in research (particularly in qualitative research) has been the use of ‘ethnically and linguistically matched’ researchers (*see* Boneham 2002; Levkoff, Levy and Weitzmann 2000; Shanley *et al.* 2013). It has been proposed that such matching is useful in research because of the researcher's presumed ‘insider’ status among the participants that they study, which in turn tends to facilitate greater trust, rapport, cultural understanding and sensitivity within research and fieldwork processes (*see* Bhopal 2001; Gallagher-Thompson *et al.* 2006; McLean and Campbell 2003). This view regarding the possibilities and usefulness of ethnic or cultural ‘matching’ in research has, however, increasingly been challenged within the more recent and growing body of literature. As with age/ing identities and cultures (*see* Arber and Ginn 1995; Heaphy, Yip and Thompson 2004; Higgs 2012), scholars researching race and ethnicity have not only pointed to the social and cultural heterogeneity existing within any ethnic category or social grouping but also emphasised the multiple, overlapping, intersecting and shifting nature of researchers' and participants' own identities, social locations and positionalities (*see* Sin 2007; Song and Parker 1995; Wray and Bartholomew 2010; Zubair, Martin and Victor 2012*a*, 2012*b*). This diversity and complexity within any ethnic category, relating to multiple social identifications, locations and positionalities, makes simplistic notions of ‘ethnic matching’ (and the perceived accompanying reductions in social distance between the researcher and the participant) in research quite problematic (Twine 2000).

An over-emphasis on ethnic matching, as discussed above, also underplays the negotiatory aspects of the researcher–participant relationship, the often precarious public/private and insider/outsider roles and positions of the researcher, and the resultant need for explicit performances of the contextually relevant social identities (*see* Griffith 1998; Sherif 2001; Zubair, Martin and Victor 2012*a*, 2012*b*). Rather than taking these complexities into account, the concept of ethnic matching presumes instead an easy alliance of values and interests between the researcher and the participant based on their perceived shared cultural identity, experiences and understandings. This also fails to take into account the powerful, and often contradictory, influence of the formalised ethical and methodological frameworks and regulations relating to both the research process and the research relationships that emerge in ‘the field’. It has been argued that these formalised, institutional frameworks and regulations within which research is routinely performed are deeply embedded in (and shaped by) existing social hierarchies and social relations, and therefore these often work to structure power imbalances and inequalities within the research process itself (*see* Huisman 2008; Truman 2003; Wilson and Neville 2009).

With the recent expansion of research ethics committees' roles, and tightening regulations on how research is conducted, an emerging body of literature addressing the ethical and methodological issues in research has focused on the perceived and experienced rigidity and inappropriateness of the ethical requirements of research (*see* Beagan and McDonald 2005; Burgess 2007; Coomber 2002; Haggerty 2004; Hammersley 2006, 2009; Stanley and Wise 2010). This literature has emphasised the powerful role of the formalised ethical and methodological frameworks of research in determining the use of specific fieldwork methods and procedures by researchers. In doing so, it has also pointed to how these official frameworks and regulations, and the assumptions that underlie these frameworks, are often incongruent with the realities of ‘the field’ for both the researcher and the participant and their own lived experiences of research participation (*see* Buckle, Dwyer and Jackson 2010; Ward 2008; Wiles *et al.* 2007).

In this paper, we draw on some of the critical methodological and ethical issues raised within the existing literature in relation to the workings of the formalised research ethics frameworks. We apply some of the insights gained from this literature, as well as the broader conceptual and methodological literature on social identities, performativity, and the researching of older age and ethnicity, to our own experiences of fieldwork with our older Pakistani Muslim participants in our Economic and Social Research Council (ESRC) New Dynamics of Ageing^2^ study: ‘Families and Caring in South Asian Communities'. In particular, we reflect on the often challenging and shifting performances of multiple public/private and insider/outsider social roles and identities in ‘the field’. In doing so, we aim to present a critique of the formalised research ‘ethics' for engaging in a routine process of ‘othering’ and disempowering of the older and ethnic minority participant in a myriad of ways, for example through: (a) using and reinforcing essentialised notions of ethnicity and older age in research; (b) playing a central role in the (re)creation of a public/private divide within the research process, disrupting and constraining the extent to which the ‘ethnically matched’ researcher can perform rapport and trust with the participant; and (c) enforcing the dominant, White middle-class (and institutionally defined), ‘ethical’ norms, standards and processes of the public forum of research within the private worlds, time and space of the participants.

## Families and caring in South Asian communities – the study aims and methods

Our study, titled ‘Families and Caring in South Asian Communities', aimed to explore the social identities, daily lives, social networks and family lives of older people from South Asian communities living and growing older in the United Kingdom (UK). In particular, its aim was to focus on these ethnic minority older people's own meanings, perceptions and lived experiences of ‘age and ageing’, ‘care’ and ‘support’, ‘the family’, ‘the community’ and ‘space and place’ within the context of their daily lives as they grew older within transnational communities in the UK. Hence, our study concerned itself not only with issues of age/ing identities and gender, and how these related to our older participants' social and familial lives and specific needs for care and support, but it also touched upon participants' particular social conceptions, uses of, and interactions within time and space. Moreover, in exploring these issues, our study aimed to focus on our older ethnic minority participants' own subjectivities and understandings. To capture our older participants' own subjective meanings and understandings, we employed qualitative research methodologies in our study – including semi-structured, in-depth, interviewing and social network mapping.

Our study sample included a socially and culturally diverse group of mostly first-generation^3^ Bangladeshi and Pakistani immigrants in the UK, both women and men, aged 48 years and older^4^ and living in a medium-sized town in the South East of England. Access to these participants was gained through a broad range of sources, including the existing social networks of the two bilingual, female, South Asian researchers in the research project^5^; local South Asian community organisations; and additionally, through frequenting the Bangladeshi and Pakistani neighbourhoods in the local town and meeting people in the local South Asian shops, mosques and other popular community meeting places. However, despite the suitably bilingual South Asian (*i.e.* Bangladeshi and Pakistani) researchers' shared ethnic origins with our participants, recruitment into the study remained slow and challenging. It improved only in the later stages of the fieldwork, and this was mainly through snowballing and referrals as our participants became more familiar with our researchers.

As we have also discussed in detail elsewhere (*see* Zubair, Martin and Victor 2010, 2012*a*, 2012*b*), the shared ethnicity of the researchers with our older South Asian participants did not preclude the need for the researchers to build a greater trust and rapport with these older participants. Furthermore, developing this trust and rapport did not merely require us to show greater sensitivity towards the needs and preferences of our participants in our use of specific fieldwork processes. In addition, as we have described in some of our earlier work in relation to the Pakistani Muslim community, it also required our Pakistani Muslim female researcher to negotiate actively, continuously and visibly an ‘insider’ status within the local Pakistani Muslim community through appropriate performances of her gendered Pakistani Muslim ethnicity. Within this earlier work, the focus of our analysis had remained mainly on the embodied negotiations of identity, trust and rapport by our younger Pakistani Muslim female researcher *vis-à-vis* our older Pakistani Muslim female and male participants,^6^ and our use of socially and culturally sensitive data collection processes and methodologies^7^ at the micro level of our interactions with our participants. In this paper, we seek to extend our focus of discussion further to take into account also the contradictions within the wider research frameworks and agendas within which research is performed, illustrating how these formalised frameworks enforce public/private dualities in ‘the field’, limiting researchers' ability to perform trust and rapport and to employ sensitive research processes.

Since the focus of our discussion in this paper is mainly the workings of the formalised ‘ethics' frameworks and regulations on the ground, it is important to mention here that our study involved gaining ethics approval from our university-based research ethics committee. Non-university-based research ethics committees, such as those linked with the National Health Service (NHS) within the UK context, are far more strict and directive in their enforcement of specific, pre-defined and standardised, ‘ethical’ rules and regulations which often pay little attention to context-based contingencies in fieldwork (*see* Truman 2003; Wiles *et al.* 2007). Despite the relative leniency of our university-based research ethics committee's specific ethical requirements in relation to our fieldwork with our older South Asian participants, we nevertheless encountered particular barriers and challenges in our fieldwork resultant from these institutionally defined ethical requirements, with their often stereotypical underlying assumptions regarding older people, ethnic minorities, participant vulnerability, and ethical conduct with respect to researching ‘vulnerable’ and socially marginalised populations. In the next part of this paper, we turn our attention to the specific social contexts of our participants and describe some of the key challenges and barriers we faced in conducting our fieldwork ethically. In doing so, we specifically highlight the tensions between the formalised ethical and methodological dimensions of conducting funded research and the realities of being in ‘the field’.

## Formalised research ‘ethics’ and public/private performances in ‘the field’

### Structuring of time in daily life

Time is a social construct whose heterogeneous character is reflected well in the multiplicities of its models, conceptions and uses across different social, cultural, situational, historical and economic contexts (Birth 1999, 2004, 2013; Duncheon and Tierney 2013; Pronovost 1986). According to Huisman (2008: 384), ‘Time is cultural, and one's perception of time and orientation to time are largely influenced by cultural norms'. She describes the conflicting nature of her own culture-specific time orientations – as a researcher and member of the dominant culture of the United States of America (USA) – with those of her Bosnian participants, and the resultant tensions arising during her fieldwork:

Under capitalism, time is viewed as a commodity – time is money and should not be wasted. In the USA-dominant culture, time is largely viewed in rigid, segmented, and linear terms. In contrast, most Bosnians I met – most of whom had lived the majority of their lives in urban locales in Bosnia under a socialist system, in which social connection often takes precedence over time – had a far more flexible orientation to time than I did. They would laugh when I would pull out my calendar to schedule our next meeting, and tell me to just come over whenever I wanted, that it was not necessary to make an appointment … I wanted to be efficient with my time and would feel frustrated when I would arrive at someone's home to do an interview and the person had forgotten or had decided to invite friends and family over or had simply used various tactics to postpone the interview. (Huisman 2008: 384)

Huisman's (2008) experiences, in relation to her own and her participants' use of time, in ‘the field’ are resonant of our own experiences of ‘time’ during fieldwork with our older Pakistani Muslim participants. In a similar way to her participants, our participants also used non-hegemonic notions of time whereby a majority did not structure their daily lives strictly around the Western hegemonic clock time or using calendars and diaries. As opposed to planning their time-use in advance or having fixed (or discrete) time-slots for various daily life activities or routines, their perception and sense of time tended to be quite ‘moment to moment’. This involved engaging in activities, and dealing with many of the social commitments, in a much more flexible and spontaneous manner as these came along. Moreover, for most of these older Muslim participants, their particular use of everyday time also revolved around the changing time schedules of the five daily prayers. However, while Huisman describes her participants' different time-use by focusing on their specific cultural and national origins in Bosnia, we would like to contend that for many of our participants their flexible use of time was also linked with their own or their close family members' particular social locations – including their statuses as retirees, unemployed or shift-workers engaged in either precarious forms of employment without fixed working hours or working unconventional hours. Hence, although in this paper we focus our discussion on the conflicting (and hence more challenging) time orientations of the majority of our participants with respect to our own fieldwork objectives and time schedules, it is important to note here also that there existed some diversity among our participants in this regard along the lines of gender, age, social class and occupation.

Huisman (2008: 385), while noting her own frustrations with her Bosnian participants' different orientations to time to herself, observes how such differentials required an adaption on her part to their time-use, lest she would be recreating within her own research the power and status hierarchies and inequalities prevalent within US society more widely, and acting as ‘yet another force in USA culture that was trying to compel them to change’. We also experienced such tensions and ethical challenges during our own fieldwork, with respect to our own differential time orientations and schedules from our participants. In our case, however, the related practical and ethical challenges were exacerbated by the specific requirements of the formalised ethical framework which bound us. First, as we were researching experiences of care and support and ageing among an older population, we were required to give our participants at least a 24-hour time gap between the provision of the information about the project and gaining their written informed consent for participation in the study. This requirement was based on ageist stereotypes and assumptions regarding older people – especially those in poor health and/or socially isolated – being a necessarily vulnerable group in research (*see* Russell 1999) which could be easily pressurised to take part unless given enough time to think through the details of the study and an easier opportunity to decline participation. More importantly, however, rather than protecting our participants or safeguarding their interests, this requirement introduced specific ethical and practical challenges in the fieldwork through enforcing (even if indirectly) Western conceptions and uses of ‘time’ within ‘the field’ and prolonging the recruitment and data collection processes for the researcher.

The majority of our participants' less structured and flexible time-use in their everyday life meant that many of the times, upon being approached by the researcher on the first occasion and being provided the study information, our participants would immediately ask ‘Can you take my interview right now?’ Many of these participants, including those who appeared keen to participate in the study, would express their difficulties in giving the researcher a later appointment to meet up another day to give their consent and be interviewed. They were often not sure about what they would be doing on specific dates and times, or when a friend or relative might stop by to meet them, and thus insisted that they wished to be interviewed straight away. More often than not, interview appointments which were pre-booked got cancelled at short notice, many times after the researcher had travelled a long distance to the participants' homes having checked over the telephone prior to making the journey. Most of the interviews, therefore, had to be rescheduled several times. Moreover, the participants would often contact the researcher rather unexpectedly via telephone and invite them to come over for an interview straight away. They would explain, for example, that a friend or some family member who was going to come to visit them that day had to go somewhere else and they were free until the time some other family member or friend might drop in to see them.

We have described in detail elsewhere (*see* Zubair, Martin and Victor 2012*a*, 2012*b*) how, in order to research sensitively and develop non-hierarchical research relationships, we focused our own efforts during our fieldwork on developing a greater trust and rapport with our participants on the basis of our Pakistani Muslim female researcher's (*i.e.* the first author of this paper) ‘shared’ ethnicity and socio-cultural understandings with our participants and her embodied cultural performances. In addition, we also tried considerably to accommodate to the different time frames and time orientations of our participants – for example, by making sure not to contact our participants over the telephone during the times of the five daily prayers and also stopping our interviews at prayer times to allow participants to pray (with our Pakistani Muslim researcher often accompanying them in the prayers), thus undoing or un-performing and disembodying the public or formalised character of our Pakistani Muslim researcher's social interactions with our participants. Within the given social context of our older Pakistani Muslim participants as described above, our insistence on booking interview appointments rather than conducting interviews spontaneously without the required 24-hour time gap nevertheless enforced a public/private divide within our fieldwork and was experienced by both the Pakistani researcher and our participants alike as being disruptive to the performance of trust and rapport in ‘the field’. This was often also commented upon by the participants, as one of our older male participants who wished to be interviewed straight away challenged our official requirements, focusing on the researcher's direct relationship with himself, and reasoned ‘If I trust you and you trust me, why wait. I'm telling you myself, I want to do it now, I don't need time to think.’ On other occasions, a few of the participants who had rescheduled their own interviews several times at short notice and/or had kept the researcher waiting for long hours at their homes before being able to give an interview, told the researcher apologetically that such delays were the reasons why they had been reluctant to fix their interview appointment in advance and had requested instead to be interviewed on the spot.

As illustrated also by Truman (2003) previously, the examples above clearly reveal how the formalised ethical requirements in relation to our research were at odds with the expectations and needs of our participants on the ground. These examples also show that, while older and ethnic minority participants have often been presented within research methodology literature stereotypically as particularly challenging populations to research, the difficulties in recruitment and data collection with these groups of participants appear to be linked with the dominant hegemonic White, working-age and middle-class frameworks and procedures employed in research with these groups. As we have shown in our case, so far as these formalised ethical and methodological frameworks are based on, and promote, essentialist notions of age and ageing as well as ethnicity, these remain typically White, young and middle-class orientated and fail to accommodate flexibly ‘difference’ within the research process. Hence, the 24-hour gap requirement that we had to contend with during our fieldwork and our own need to pre-book interview appointments, effected long delays for us in our data collection, rather than the particular old age vulnerabilities of our participants or their specific ‘cultural’ differences in time-use from the White, young and middle-class norm. Even as our participants often tried to accommodate our difference within their own time schedules, despite our eagerness to learn about our participants' own lives, our formalised ‘ethical’ and procedural requirements often worked to impose the norms of the public forum of research into the private spaces and times of our participants.

### Gendered use of public and private spaces

One of the key challenges that we faced in our research was in relation to using the Pakistani Muslim female researcher's ethnic identity for enhancing our fieldwork, coupled with our choice of an informal style of conducting fieldwork within the gendered public and private spaces of our participants (*see* Zubair, Martin and Victor 2012*a*, 2012*b*). These particular choices were linked with both the perceived and experienced difficulties of recruitment of older South Asians – particularly the women – into the study, but also our own inclination towards engaging more with our study participants and hence resisting to some extent the reinforcement of structural inequalities in our own fieldwork or, as Irwin (2006) aptly points out, ‘doing structure’ in the field.

‘Ethnic matching’ of researchers to their participants, particularly in research with ethnic minority populations, has indeed often been perceived positively with underlying assumptions regarding the equalisation of the otherwise hierarchical research relationships between researchers and their participants. On ethical grounds, ethnic matching may also be more commendable for allowing greater access to, and hence inclusion of, minority and socially marginalised voices and perspectives into research. Boneham (2002), for example, has noted how older South Asian women's voices and experiences often remain hidden within research because male community leaders and family members become important intermediaries, who speak on behalf of their female family members. Boneham, therefore, proposes bridging the gap between the ‘private’ worlds of the older South Asian women and the White public forum of research through inclusion of female researchers of the same cultural background as the older women within the research process. Our own experience of fieldwork with our older Pakistani Muslim participants also reveals the important role of the Pakistani Muslim female researcher's ‘shared ethnicity’ in navigating the gendered community public and private spaces and negotiating access into the women's, as well as the men's, private worlds (*see* Zubair, Martin and Victor 2012*a*, 2012*b*). However, as we illustrate below, such an emphasis on a shared ethnicity within the gendered spaces of our participants masks the numerous tensions and ethical dilemmas experienced by the researcher and the participants alike, specifically in relation to the performances of their public and private identities and positionalities.

We have discussed in detail in some of our earlier work (*see* Zubair, Martin and Victor 2012*a*, 2012*b*) the risks and vulnerabilities that were created during our fieldwork for the ‘ethnically matched’ Pakistani female researcher because of her precarious status within the Pakistani Muslim community as being simultaneously both an insider and an outsider. We also illustrated how such vulnerabilities were exacerbated for the researcher because of the gendered use of space within the community and her need to access and recruit also the older men in their separate, gendered, community spaces. This often led to difficulties in navigation for the researcher between the women's and the men's separate spaces, and also uneasy performances of her public/private and insider/outsider identities and statuses within the community. This was because, while an emphasis on a shared ethnicity was useful in gaining access to the women's spaces, this ‘shared’ yet gendered ethnicity denied her easy access to the men's spaces. Successful navigation within the men's spaces required instead a cautious interplay in performances between the researcher's ethnic, personal/familial and public (or professional) identities and roles. With the progression of time spent by the researcher in ‘the field’ and her greater immersion with the local Pakistani community, particularly with respect to the women's gendered spaces, the researcher's identity as a gendered ethnic insider increasingly gained prominence over her more public-oriented, professional identity as a researcher. This meant that even though the official research protocols for the researcher's safety in the field as a lone worker remained in place, and were adhered to by the research team, negotiating her presence and engagement within the male spaces became challenging as many of her key contacts and participants within the community, perceiving of her as a younger woman from the community, advised her not to approach men or interview them alone. On other occasions, the researcher was also exposed to considerable social pressure to accept private invitations to the women's social activities at their homes, mosques and community centres. These invitations were often difficult to decline, especially where the female contact or participant had gone to considerable lengths to help the researcher with recruitment for the study. Such examples of the experiences of the ethnically matched researcher in relation to avoiding ‘doing structure’ in the field illustrates very well some of the complexities and practical and ethical tensions inherent in the management and performance of varied social locations and self-positionings on the ground by researchers – whether ethnically matched or not. Researchers often need to contend with such lived fieldwork challenges, which are not easily addressed through formalised research ethics frameworks, making practical and ethical judgements independently and case by case.

Our own experiences of fieldwork in relation to gaining access to gendered community spaces, using an ethnically matched researcher as described above, thus highlight the importance of being mindful of the complex dynamics of the dual public/private character of most research relationships. In a similar way to the Pakistani Muslim researcher who was conducting the fieldwork, our older Pakistani Muslim participants also appeared to experience the research encounter as being of both a public and private character, and hence often shifted their performances between their own public and private identities. As found previously by Russell (1999) in relation to fieldwork with socially isolated White older people, rather than being vulnerable older people, many of our older ethnic minority participants – including the women – resisted the perceived and actual public invasion through research in their own private spaces by exercising considerable control over both the character of their relationship with the Pakistani Muslim researcher and also what they disclosed in their interviews.

All participants and research contacts more generally, but particularly the women, in their interactions with the female Pakistani researcher conducting the fieldwork focused more on the personal and ‘private’ aspect of their relationship with the latter based on her identity as another female member of the local Pakistani community. Hence, they would often invite the researcher to their homes for tea, introduce her to their family and friends, and ask her to keep visiting, but many appeared reluctant to participate in the study during the earlier phases of the fieldwork. Those women who did agree to participate in interviews appeared to give primacy to the researcher's co-ethnic role within their private spaces and negotiated rapport through a negation of the encounter as being primarily a research encounter. They would, therefore, often take control over the researcher's time, insisting to her, for example, to first have tea with them before ‘getting the interview out of the way’. During the tea, they would engage in long conversations with the researcher about the latest news in relation to some family member or friend, tell her about some new recipe they had learnt, show her some new clothes they had bought for some wedding or show a family wedding video, ask her for her advice on issues to do with their children's or grandchildren's marriage or schooling, or even offer her some form of personal help they perceived she could need herself such as finding more suitable accommodation in the local area. In this respect, they seemed to be renegotiating and making meaning of the research encounter within their private spaces as a personal encounter of reciprocity or help between co-ethnics.

In many cases, the informal and ‘private’ character of the research encounter would, however, suddenly change during the actual course of the interviews when some of the participants (including both the women and the men) would become quite cautious in their responses, readjusting their levels of disclosure about themselves and their own families and personal lives during the interviews, thus shifting to the more ‘public’ performances of their own identities and personal selves. One male participant, for example, during his audio-recorded interview consistently kept rephrasing what he told the researcher, sometimes covering the microphone with his hand and at other times whispering to the researcher, identifying certain parts of the interview conversation as information that he was only disclosing to her and wished to be deleted from the rest of his ‘official’ interview. Not very surprisingly, his shifting private and public performances of the self – when doing rapport and trust with the researcher through personal disclosure in private, and yet presenting a positive public image of the Pakistani community – were apparent in his insistence to edit out from the interview certain disclosures and personal opinions which presented a negative view of his family and the Pakistani community to the outside world. This illustrates how the actual concerns and lived experiences in relation to research participation appeared to be much different for our older ethnic minority participants to those defined by the formalised research ethics. Despite being ethnic minority older people, our participants (including the women) were neither the vulnerable ‘others' who could be coerced into research participation and personal disclosure without prior adequate knowledge or understanding of what it incorporated, nor were they necessarily a ‘hard-to-reach’ social grouping with silenced voices and unable to navigate the research encounter tactfully on their own terms. As we discuss further in the remaining paper, the challenges associated with involving this group of older people in research were linked more with the formalised ethical and methodological frameworks and procedures of research employed and dictated by White middle-class and bureaucratic institutions which routinely engage in *othering* processes in relation to research participants more generally, and are often experienced particularly negatively by ethnic minority and other socially marginalised research populations.

### Orality of informal social contexts and relationships

An important aspect of researching ethically from the standpoint of institutionalised ‘ethics', as enforced by most research ethics committees, involves the process of gaining ‘informed consent’ from participants. In the case of our study, this involved providing our potential participants with written participant information sheets giving ‘sufficient details' about our research project and what participation involved, and obtaining participants' written consent to participation on our standardised, official consent forms relating to the study and endorsed by our university. As a measure to ensure equal access to study information for all potential older Pakistani participants, and hence a fair inclusion of a range of diverse voices and perspectives in the research study, we produced our participant information sheets and consent forms in both English and Urdu. While this pre-defined and agreed ethical requirement of gaining a formal, written, informed consent addressed the concerns of our institutional research ethics committee, it presented us and our research participants with particular challenges and concerns during the fieldwork, often bringing into question the ethical legitimacy of our informed consent process.

Even though we provided our participants with participant information sheets in advance and gave them at least 24 hours to decide if they wanted to participate, in most cases the participants did not read the information sheets at all prior to their interviews. While some of the older women who had had no schooling were unable to read information provided in Urdu either, this issue was far from being linked merely with our participants' particular literacy or linguistic skills. This was quite apparent to us as a large majority of those participants who had the required literacy and linguistic skills to be able to read the information also often did not read it. Many of the participants asked the Pakistani researcher to tell them about the content of the information orally herself or to read out to them only those parts of the information which she deemed were important. Some of the participants also insisted that they were not interested in reading or hearing the information, but as they trusted what she had already told them about the research project, they were happy to be interviewed on that basis.

Getting written informed consent from potential participants, in the form of a signature on an official consent form, presented us with particular challenges. Being in stark contrast to the oral character of most informal social contexts and relationships, including those within which we were conducting our own fieldwork, the introduction of written information sheets and consent forms made the process much more formal and official (*see* Wiles *et al.* 2007). Hence, rather than serving as a form of assurance for the participants with respect to their own protection within the research process, it was experienced as threatening by many of our participants who expressed their concerns about having to sign a paper which they thought seemed to be unnecessary. Many of them questioned why the research team needed their name and signature on an official form if information in relation to their identities was really going to be kept confidential and not used at all. Such concerns became very obvious to us when one participant decided to withdraw from the research when he was only 15 minutes into his interview, as he was beginning to feel uncomfortable about having signed his name on an official paper. A couple more participants, after having given their interviews, requested the Pakistani researcher to not reveal any of the ‘evidence’ against them if she came across it later when writing her report. While they insisted that they did not wish to withdraw from the study, they revealed their discomfort with the written consent procedure by suggesting that they had given their interviews as a form of help to her and her research project team and so they trusted that she and her research team would also reciprocate by not revealing in their written official report anything that could be potentially harmful for them.

The concerns among our older Pakistani participants in relation to the potential compromise of their anonymity and confidentiality through the written official paperwork, involved as part of the ‘informed consent’ process, are neither specific to them as older ethnic minority participants nor unreasonable. Researchers working with other socially marginalised, minority or deviant groups, or in certain other social contexts, have noted similar concerns arising from the use of the official written consent process where participants could ‘fear that signed consent forms could make the information they provide traceable to them’ (*see* Coomber 2002; Wiles *et al.* 2007: 3.17). In the case of our research participants, their self-perceived and actual social positioning and location as post-colonial, largely working-class immigrants growing older in a Western welfare state, and perceptibly their earlier experiences of White officialdom and exclusions within their host country, are likely to be influential in how they interact with and experience such formal and officialised procedures. One participant, for example, upon learning that he would need to sign a consent form to participate in the study, expressed his suspicion about the research by asking the Pakistani researcher questions relating to the research funders at length and then hinted at the perceived *othering* of ethnic minorities and immigrant (as well as older) populations within the wider research agendas through his comment: ‘Why are they interested in older South Asians? They're not going to start sending people back to their countries are they, the older ones that are ill?’ While this participant's particular concerns relating to the written informed consent process are suggestive also of a general unfamiliarity with (Western) formalised research procedures (*see* Zubair, Martin and Victor 2010) shared with many other research populations, these concerns nevertheless simultaneously represent his suspicion and mistrust of the research agendas based on his identity as an older ethnic minority immigrant.

In some of our earlier work (*see* Zubair, Martin and Victor 2012*a*, 2012*b*), we have illustrated the importance of the researcher's presentation of their embodied ‘self’ in gaining participant trust through appropriate performances of their ‘insider’ co-ethnic status. In particular, we highlighted how the Pakistani researcher who was conducting fieldwork with our older Pakistani participants managed to negotiate insiderness within the Pakistani community through her appropriate use of language, dress, bodily performances, and appropriate use of time and space within the informal and ‘private’ social contexts of our participants. We contend here, however, that the performativity of this embodied trust and rapport by the researcher within the ‘private’ worlds of our participants was often disrupted during our fieldwork as a result of the researcher's intertwining performance of her public and official role, embodied within the act of presenting and using the official paperwork – including the signing of an official contract between the research team or the academic institution and the participants in the form of the written consent forms. This act was often experienced by both the researcher and the participants alike as positioning them within the formal public realm but on the opposite ends of the officialised ‘us' and ‘them’ divide. Hence, often perceiving the written consent process as protecting institutional interests rather than their own (*see* Truman 2003), many of the potential participants and contacts would make comments to the researcher, such as ‘But what's the benefit in all this for us?’ Some of the participants pointed out to us that they were already giving help to us by agreeing to be interviewed, having to sign an official form to do so was perceived and experienced as an extra burden placed on them. Many others resisted the power-imbalance created within this formalised research encounter, suggesting to us that they would be happy to participate if they could be interviewed without signing a consent form, while a few others who saw the interview as an opportunity to talk about their lives freely with the interviewer expressed feeling excluded as a result of the formal ‘informed consent’ requirement.

## Concluding comments

Recent scholarship on research methodologies and ethics has begun to point strongly to the incongruities that often exist between formalised research ethics frameworks and regulations – enforced upon those undertaking funded academic research, and research participants' own perspectives and lived experiences of research (*see* Buckle, Dwyer and Jackson 2010; Truman 2003). Truman (2003), for example, has identified formalised ethical guidelines and regulations as protecting institutional interests without necessarily addressing the moral obligations of researchers, and thus introducing inequalities within the process of research production itself. Other scholars (*see* Coomber 2002; Hammersley 2006, 2009; Stanley and Wise 2010) have further commented on the contradictions that lie at the heart of the actual workings of formalised research ethics regulations and structures, such that these ‘rupture the relationship between following the rules and acting ethically’ (Haggerty 2004: 391). In this paper, focusing upon issues of time and space use and the oral character of informal social contexts and relationships, we have attempted to illustrate some of these contradictions as we experienced them during our own fieldwork with our older Pakistani Muslim participants living in the UK. In particular, we have highlighted three key processes with respect to the potential *othering* and disempowering of older and ethnic minority participants within the existing formalised research ethics frameworks. First, we have pointed to the use and reinforcement of essentialised notions of older age and ethnicity in research which often negatively position older and ethnic minority people in terms of their vulnerability and cultural difference, and hence as particularly challenging populations to research. Second, we have shown how the recreation of a public/private divide within the research process through the use of formal procedures reinforces hierarchical research relationships, disrupting and constraining the ability of researchers and their participants to perform rapport and trust within the research relationship. Third, we have illustrated the potential for power imbalances and structural inequalities in the research relationship, arising from ‘doing structure’ in the field simply through the use of institutionalised ‘ethics' codes – particularly when White, middle-class and institutionally defined ethical standards and practices of the public forum of research are given priority over older ethnic minority research participants' own concerns and employed in standardised ways to the disadvantage of those being researched within their own private worlds.

In our reflections relating to our own fieldwork experiences, we have placed at the centre the specific social context of our participants in their self-perceived and actual social positioning and location as post-colonial working-class immigrants growing older in a Western welfare state. Focusing on the perspectives and experiences of these older ethnic minority people in relation to their role as research participants, we have illustrated that many of the challenges and barriers associated with conducting research and fieldwork with this group of older people can be explained in terms of the public/private social and cultural divide that is inherent within the formal research procedures and ethical frameworks employed as part of doing funded research. We use this premise to further argue here that while it is very important to develop greater social and cultural understandings of this under-researched group of older people during the research process, it is also equally important to shift our gaze away from a sole focus on the often over-emphasised social and cultural differences of these ethnic minority older participants. Drawing parallels with the situations of other socially and culturally marginalised research populations which share similar situations and concerns in relation to participation in research, we argue that instead of focusing on the problems associated with the perceived older age and ethnically based cultural differences and norms of these and other ethnic minority and older research participants, it is more fruitful to turn our attention towards the actual frameworks within which research and fieldwork is performed. More specifically, we argue for the need for a more systematic and thorough consideration of how the actual frameworks made available to researchers and research teams for conducting research, and the agendas and processes employed and dictated by White, middle-class and bureaucratic institutions, may be perceived by certain research populations as putting them in a particularly vulnerable or less desirable position. Finally, it may be useful to reconceptualise and reflect on good ‘ethical’ practice in qualitative research with older ethnic minority people differently – as an ongoing, case by case, consideration of the concerns of the research participants themselves within the often dynamic and situational context of fieldwork and ‘field’ relationships, rather than a mere adherence to formalised procedures and codes of practice (*see* Burgess 2007; Dequirez and Hersant 2013; McDonach, Barbour and Williams 2009; Small 2001; Wiles *et al.* 2007).

## NOTES

1 Our own study, while not using participatory approaches in the strictest sense, adhered to some of the principles of participatory research – *e.g.* through the inclusion of a local advisory board within the research process, comprising local South Asian community leaders.

2 The ESRC New Dynamics of Ageing programme was an eight-year multi-disciplinary research initiative in the UK with the aim ‘to develop practical policy and implementation guidance and novel scientific, technological and design responses to help older people enjoy better quality lives as they age’. The programme involved integrating understandings of the changing meanings, representations and experiences of ageing, and the key factors shaping them, through direct engagement with older people and user organisations (*see* http://www.newdynamics.group.shef.ac.uk/about-the-programme.html, accessed 16 February 2014).

3 Most of our participants were first-generation immigrants in the UK. However, there were also a few participants who may more appropriately be described as being one and a half generation because of having migrated to the UK as children or adolescents under 18 years of age.

4 The inclusion of relatively ‘younger’ ages in our sample (*i.e.* ranging from 48 years onwards) in comparison to the existing, dominant, definitions in the literature on ‘older adults' reflects not merely the younger age profiles of these ethnic minority populations in the UK (particularly the Bangladeshi population) but also our participants' own subjective perceptions, conceptualisations and categorisations of older age as revealed in our study data.

5 While, in this paper, we focus mainly on the fieldwork experiences of our Pakistani Muslim researcher with our Pakistani Muslim older participants, it is important to mention that our research project team comprised four members in total – a Principal Investigator and a Co-Investigator who were both White British women and two female research fellows of South Asian descent.

6 We discuss issues relating to our first-generation Pakistani Muslim female researcher's own younger age when researching our older Pakistani Muslim participants in another paper (*see* Zubair, Martin and Victor 2012*b*).

7 Our employment of specific data collection and fieldwork processes in our study is discussed in more detail in another paper (*see* Zubair, Martin and Victor 2010).
